# Predicting Length of Stay and Discharge Destination for Surgical Patients: A Cohort Study

**DOI:** 10.3390/ijerph17249490

**Published:** 2020-12-18

**Authors:** Fabrizio Bert, Omar Kakaa, Alessio Corradi, Annamaria Mascaro, Stefano Roggero, Daniela Corsi, Antonio Scarmozzino, Roberta Siliquini

**Affiliations:** 1 Department of Public Health Sciences, University of Torino, 10126 Torino, Italy; fabrizio.bert@unito.it (F.B.); omar.kakaa@unito.it (O.K.); annamaria.mascaro@edu.unito.it (A.M.); roberta.siliquini@unito.it (R.S.); 2 Department of Quality and Safety of Care, A.O.U. City of Health and Science of Torino, 10126 Torino, Italy; sroggero@cittadellasalute.to.it (S.R.); dcorsi@cittadellasalute.to.it (D.C.); ascarmozzino@cittadellasalute.to.it (A.S.)

**Keywords:** discharge planning, surgery, cohort study, early prediction, difficult discharge, length of stay

## Abstract

Discharge planning is important to prevent surgical site infections, reduce costs, and improve the hospitalization experience. The identification of early variables that can predict a longer-than-expected length of stay or the need for a discharge with additional needs can improve this process. A cohort study was conducted in the largest hospital of Northern Italy, collecting discharge records from January 2017 to January 2020 and pre-admission visits in the last three months. Socio-demographic and clinical data were collected. Linear and logistic regression models were fitted. The main outcomes were the length of stay (LOS) and discharge destination. The main predictors of a longer LOS were the need for additional care at discharge (+10.76 days), hospitalization from the emergency department (ED) (+5.21 days), and age (+0.04 days per year), accounting for clinical variables (*p* < 0.001 for all variables). Each year of age and hospitalization from the ED were associated with a higher probability of needing additional care at discharge (OR 1.02 and 1.77, respectively, *p* < 0.001). No additional findings came from pre-admission forms. Discharge difficulties seem to be related mainly to age and hospitalization procedures: those factors are probably masking underlying social risk factors that do not show up in patients with planned admissions.

## 1. Introduction

Discharge planning is the process of tailoring, in advance, individual paths for patients regarding their discharges. A recent Cochrane review showed that discharge planning reduces hospital lengths of stay and readmission rates (−0.73 days and 0.87 OR, respectively) [1]. However, there is a lack of data about surgical patients. In the same review, published in 2016, only five studies reported surgically related data. However, interest in as-fast-as-possible recovery after surgery is not novel [2,3]. More recently, the Enhanced Recovery After Surgery (ERAS) program was developed, in order to improve not only the speed of recovery but also quality [4]. Indeed, discharge planning can also increase the satisfaction of both patients and healthcare professionals [1].

Many frameworks have been developed to better describe the factors associated with the discharge of surgical patients. One of these, by Morgan and Beech, divides those factors into four main categories, connected to each other: the characteristics of the patients admitted, the characteristics of healthcare systems, clinical practice styles, and the organization of hospital care [5]. In this model, all these variables concur to determine the total length of stay (LOS), and additional clinical, social and economic outcomes. Focusing on the characteristics of the patients admitted, many tools have been developed to support discharge planning. Mainly, these tools try to predict the likelihood of complications during the hospitalization. Furthermore, these tools try to predict functional adverse outcomes, which can pose serious difficulties during the discharge process [6]. Most of these tools are appropriate for patients admitted for medical conditions, and the majority of those are condition-independent and can be widely applied. However, there is a lack of tools tailored for surgical patients. Even though some scoring algorithms have been developed, these are very specific and suited for a single type of intervention. Some examples are the Risk Assessment and Prediction Tool (RAPT), for hip and knee arthroplasty surgery [7], and the Parsonnet score, for surgery in acquired adult heart disease [8]. Other tools are indeed suited for the unspecific surgical patient, such as the POSSUM (Physiological and Operative Severity Score for the enUmeration of Mortality and Morbidity) score [9]. However, these tools require the collection of intervention items, such as total blood loss, and cannot be used before surgery. This characteristic hinders the possibility of using these scores at the admission phase, such as for BRASS in emergency department settings [10], or even earlier, at the pre-admission phase.

Furthermore, surgical patients often need assistance that they did not need before, either by the loss of previously achieved autonomies or by the gain of a new disability (e.g., in the event of a stomia). This kind of patient is more sensitive to a scarcity of social support, often needing longer healthcare assistance in the case of the absence of caregivers. However, only some of the tools utilized assess this kind of social vulnerability.

For all these reasons, more data are needed about those factors already present before proper intervention associated with a longer length of stay or a discharge with the need for additional care, and that can be investigated in the early phases of the surgical pathway.

The aim of this study was to investigate those factors in a large sample of patients, in order to better understand what can be done to predict, as early as possible, which patients will need personalized and more-demanding discharge planning, and possibly to suggest general items suitable for this prediction in general surgery patients.

## 2. Materials and Methods

### 2.1. Study Design

A retrospective cohort study enrolling surgical patients was performed in one of the largest hospitals in Northern Italy. Data were retrospectively collected from hospital records (SDOs in Italian), acquiring personal and clinical data of patients undergoing general surgery procedures, in a sample spanning January 2017 to October 2019 (Period 1). Furthermore, other data were collected from a smaller sample of patients undergoing pre-admission visits, from November 2019 to January 2020 (Period 2). During these visits, an ad hoc questionnaire was completed, in addition to the questionnaires routinely performed, which collected more clinical and personal data, with a focus on social vulnerabilities. Thus, the Period 2 database included only those patients for which a pre-admission questionnaire was available. Different analyses were performed on the Period 1 database and the Period 2 database. All the procedures performed were in accordance with the 1964 Helsinki declaration and its later amendments. The study was approved by the Institutional Review Board of the Department of Public Health Sciences and Pediatrics.

### 2.2. Data Collected

The variables collected from the SDO database were the following: sex, place of residence (categorized as regional, extra-regional, or foreign), hospitalization procedure (Emergency Department, planned admission, or other), neoplasia diagnosis as the main diagnosis, number of transfers, discharge destination (dead, no need for additional care, or need for additional care), Diagnosis Related Group weight (≤1 versus >1) [11], Diagnosis Related Group—complications (DRG with complications/comorbidities versus DRG without complications/comorbidities), age, and LOS. Then, in the subset of cases with a completed pre-admission form, other variables were collected, namely, neoplasia presence at the pre-admission visit, smoking status (smoker versus non-smoker), presence of a caregiver, living alone (yes versus no), at least one fall in the past year, and the need for a walking aid. In the statistical analysis, neoplasia presence was intended as a “main diagnosis” in Period 1 and as “known at pre-admission” in Period 2.

The main outcomes were the length of stay and discharge destination.

### 2.3. Statistical Analysis

Descriptive analyses were performed for all variables. Age, LOS, and BMI were treated as continuous variables and are described with medians and interquartile ranges (IQRs) because of non-normal distributions (Shapiro–Wilk test and Kolmogorov–Smirnov test). The remaining categorical variables are described as frequencies and percentages.

The LOS was then analyzed in variable subgroups (e.g., males vs. females) in the Period 1 dataset. Subgroup differences were assessed with the Mann–Whitney U test and Kruskal–Wallis test for categorical variables, and with univariable linear regression models for continuous variables. Differences in the variable distributions in association with the discharge destinations were assessed with contingency tables and chi-square tests if the variable was categorical, and with Kruskal–Wallis tests if the variable was continuous.

Multivariable linear regression models were developed to weigh the associations of factors with the main outcomes. First, the Period 1 database was analyzed. A linear regression model was fitted, with the LOS as an outcome. The same model was fitted another time but excluding those variables not available at admission time. Then, a logistic regression model was used to find associations between variables and discharge destinations. In this case, deceased patients were excluded from the analysis, achieving a dichotomous outcome, that is, “no need for additional care” versus “need for additional care”. Additionally, in this case, a second model was fitted exclusively including the variables available at admission time.

Then, the Period 2 database was analyzed with similar models, with the added variables from the pre-admission form. In this case, the models were fitted only including the variables available at pre-admission and admission.

All independent variables were chosen a priori based on the expected relevance.

The results are expressed as unstandardized coefficients (Bs) for linear regressions and as ORs (expBs) for logistic regressions. The SPSS software (version 25) (IBM Corp., Armonk, NY, USA) was used, and a two-tailed *p*-value < 0.05 was considered, with 95% confidence interval (95% CI) calculation. Missing values were excluded pairwise for descriptive analyses and listwise for others.

## 3. Results

### 3.1. Characteristics of the Sample

A total of 15,165 SDOs were collected during Period 1. Of these, males made up 7935 (52.3%). The median age was 62 (Shapiro–Wilk normality test *p* < 0.001), with great variability (IQR, 22). The grand majority (90.4%) of the patients came from the Italian region of the hospital, while 9.5% came from another region, and only 28 (0.2%) came from abroad. Regarding hospitalization procedures, most patients (68.9%) followed a planned path, 18.7% came from the Emergency Department, and the remaining (12.3%) were hospitalized in other ways (e.g., transferred from another hospital). A good number of patients (3883, 25.6%) had neoplasia as their main diagnosis. More than half of the sample (53.1%) had a DRG weight more than 1 [11], and only 18.9% had a DRG classified as “with complications”. Looking at the outcomes, the median LOS was 3, with an IQR of 6, a mean of 6.37, and an SD of 9.81 (Shapiro–Wilk normality test *p* < 0.001). Finally, the grand majority (97%) of the sample did not need any additional care after discharge, 2.5% needed it, and 0.5% became deceased. In Table 1, the descriptive variables are shown, stratified by subgroups. Mainly, the LOS was different in almost every subgroup, with a great difference when grouping patients by hospitalization procedure. The same variable was strictly associated with the discharge destination.

Regarding Period 2, while 2278 SDOs were collected, only 388 pre-admission forms were retrieved: Period 2 analyses were performed on this sample. Of the patients, 54.1% were male, and the median age was 61 (IQR, 20). The median BMI was 26.12, with an IQR of 6.9 (Shapiro–Wilk normality test *p* < 0.001). Of the individuals, 56.4% were married and 18.3% were unmarried. Additionally, 95.4% came from the same Italian region of the hospital, and 90.2% followed a planned hospitalization procedure; only 1.8% were hospitalized in the Emergency Department. Smokers accounted for 21%. A proportion of 17.8% had a known neoplasia at the pre-admission visit, and 47.9% had a DRG weight greater than 1 at discharge time. Falls during the preceding year had been experienced by 10.4% of the sample, and 2.9% needed a walking aid. Finally, 96.1% of these patients were declared to have a caregiver, but only 82.4% lived with other people, the remaining 17.6% living alone. Furthermore, only 68.8% had a caregiver younger than 65, while 31.2% had an older caregiver or did not have a caregiver at all. In this sample, the median LOS was 2, with an IQR of 3; 99.2% were discharged at home, without the need for additional care. Full descriptive data are available in Table 1.

### 3.2. Factors Associated with an Increased LOS

The first model assessed all the variables associated with a longer LOS. Almost all the entered variables showed statistical significance. In first place, having patients discharged without the need for additional assistance as the reference category, patients discharged with additional care needs had an increased LOS of 10.76 days (*p* < 0.001), while deceased patients had an increased LOS of 9.78 days (*p* < 0.001). The second factor by importance was the hospitalization procedure: having a planned path as a reference, patients hospitalized in the ED had an increased LOS of 5.21 days, while the “Other” category had an increase of 4.32 days (*p* < 0.001 in both cases). Looking at clinical factors, patients with a DRG weight greater than one had a longer LOS by 4.32 days, while a DRG with complications increased the LOS by 3.68 days. Finally, a diagnosis of neoplasia increased the LOS by 2.57 days. A *p*-value < 0.001 was found for all these associations, too. Then, every year of age increased the LOS by 0.04 days (*p* < 0.001). A significant association between sex, place of residence, and LOS was not able to be found. This model achieved an adjusted R squared of 0.289.

Looking at the model that took into account only the variables available at admission, a greater effect was found for the hospitalization procedure. Patients admitted through the ED had an increased LOS of 6.92, while the “Other” category had 6.44 days of increased LOS (*p* < 0.001 in both cases). A new association was found for place of residence, with patients coming from other regions having 0.93 days of increased LOS in comparison to patients coming from the same region of the hospital (*p* < 0.001). Each year of age increased the LOS by 0.08 days, and also, in this case, a statistically significant association between sex and LOS was not able to be found. In this case, the adjusted R squared was 0.119.

Finally, the third model added variables collected during pre-admission visits. Additionally, in this case, the hospitalization procedure had a greater effect. Patients admitted through the ED had an increased LOS of 8.22 days (*p* < 0.001). A diagnosis of neoplasia was responsible for an increase of 3.49 days (*p* < 0.001), while each year of age increased the LOS by 0.03 days (*p* = 0.017). A significant association was not able to be found for the added variables, namely, smoking status, living alone status, at least one fall in the last year, need for a walking aid, and presence of a young caregiver. Furthermore, no association was able to be found between sex, place of residence, and LOS. The model had an adjusted R squared of 0.284.

All the results about the LOS-associated variables are shown in Table 2.

### 3.3. Factors Associated with Discharge with Need for Additional Care

The first logistic regression model assessed the associations between the variables and discharge with a need for additional care. The main association was found with the hospitalization procedure. Having a planned path as a reference category, admission by the ED increased the probability of additional care needs with an OR of 1.77 (*p* < 0.001). Patients admitted through “Other” ways had an OR of 2.68 (*p* < 0.001). Having a DRG with a weight greater than 1 increased the probability of a discharge with additional care needed with an OR of 1.65 (*p* = 0.001), while a DRG with complications increased it with an OR of 2.84 (*p* < 0.001). A patient with a neoplasia had an increased OR of 1.24, although statistical significance was not reached (*p* = 0.076). Finally, each year of age increased the probability of additional care needs with an OR of 1.02 (*p* < 0.001). No association was able to be found with sex and place of residence.

The second logistic regression model, evaluating only the variables available at admission time, showed similar results. At first, admission through the ED increased the probability of additional care needs with an OR of 2.54, while “Other” admittance ways had an OR of 3.39 (both *p* < 0.001). Each year of age was responsible for an increase with an OR of 1.03 (*p* < 0.001). Additionally, in this case, no association was able to be found with sex and place of residence.

For this outcome, no regression was able to be modelled for the pre-admission subsample, because in this, the frequency of patients discharged with additional care needs was very low (0.5%).

All the results about the associations between variables and discharge destinations are shown in Table 3.

## 4. Discussion

### 4.1. Discussion of Findings

This study aimed to identify factors already present at the pre-hospitalization visit that can predict which patients will have longer LOSs and the ones in need of additional care at discharge. Predicting the LOS has always been a relevant issue in hospital management [12]; many have also tried to analyze and summarize the factors that could influence the LOS, such as in the framework presented by Morgan and Beech [5]. Those topics have been the object of reviews [13], and also, governments have tried to tackle this issue, creating useful toolkits for professionals [14]. With the development of machine learning and neural systems, they have also been used and applied to predict the LOS [15], and this will be even more common in the future. Moreover, the recent year showed us how in stress situations such as COVID-19, good bed management may help the endurance of the healthcare system [16], so understanding those factors is crucial. In a broader context, the analysis of these factors may also help the management of surgical procedures that are expected to increase dramatically in the next decade, especially in low–middle income countries that are already facing a scarcity of resources and a need for good management. In this direction, the early identification of these factors could optimize the flows of surgical patients, improving the quality of the care provided and the efficiency of the system by reducing any inappropriate days of hospitalization unrelated to the clinical assistance needed during hospitalization.

Our study again confirms the role of patients’ ages as a factor involved in determining a longer LOS, as shown as well by Lin et al., and the need for additional care after discharge, as per the recent review of Kobewka et al. This correlation has also been acknowledged by several Italian scientific societies of anesthesiology, gerontology, geriatrics, and surgery that, in a recent consensus paper, recommended taking into consideration age as a key factor in perioperative management [17,18]. The role of age may also hinder, under his umbrella, several issues such as clinical problems (e.g., frailty [19] and cognitive decline) and social issues (e.g., weak social networks and the absence of caregivers), which are both tied to the LOS, as shown by Morgan and Beech [5]. For this reason, it is recommended to plan in advance a discharge plan with different healthcare professionals [17]. Regarding the social issues, although other work, such as the review by Lin et al., emphasizes the role of the presence of a caregiver [18], our study does not highlight, in Period 2, their role in the LOS and the need for additional care after discharge. Even if this may be viewed as a weak point of our study, in an operative way this finding may become advantageous, as age can be considered a proxy variable of all the physical and social factors that are the actual obstacles to discharge for the patients. In this regard, the work of Morgan and Beech is a useful conceptual framework for describing and analyzing the factors associated with the LOS, but often, it is not possible to collect all these variables for all surgical patients. More should be done to find a group of key variables able to provide an acceptable prediction.

Related to the clinical factors, our study suggested different points to look at, the first and most relevant being the type of hospital admission. Being admitted in an emergency showed the greatest impact on the LOS and on the need for additional care; the role of the emergency setting was already discussed by Havens, showing an increased morbidity and mortality in those patients [20]. Elective surgery patients seem to be less prone to encountering a prolonged LOS, also due to social issues, probably because they have time to prepare by finding support in their social network, while ED patients often need help in the discharge process, as suggested in the paper of Achanta [21].

The other clinical aspects that emerged as relevant factors affecting the LOS were DRGs with higher weights and DRGs with comorbidities and complications. These two elements can represent a proxy for the burden of the surgical operation, the comorbidities of the patients, complications related to the surgery, and the clinical needs of the patient. The relevance of those factors was also brought up by a study on the NSQIP database by Feeney et al., which highlighted the role of the procedure itself, suggesting stratifying the procedures by risk. The role of complications has also already been examined in the past [22], showing an important role, through relapses, in hospitalization costs, as shown by Pirson et al. [23].

The last clinical factor that emerged from our study was the association with neoplastic conditions: our study suggested a longer LOS for these patients. The associated frailty and possible extra needs of these patients as suggested by Shahrokni et al., should be taken into account and assessed with specific tools [24].

In summary, it can be hypothesized that patients with longer LOSs and needs for additional care after discharge have some features that can lead to early identification. The results of this study seem to suggest that social factors that were indeed demonstrated by other work are present, but not in the subpopulation that follows a planned path. Probably, those patients reach a pre-admission path because they already have stronger social support or personal resources, and/or even in the presence of social vulnerabilities, they have time to prepare for their hospitalization and subsequent discharge. By contrast, it can be hypothesized that social vulnerabilities must be assessed early in ED patients, both because it is more likely that these patients suffer from these vulnerabilities, and because if those are indeed present, there is reduced time to prepare for exposure to the augmented risks of a longer LOS and additional need for care after discharge.

Optimizing the discharge process is a well-known issue, but its importance is of rising interest, and this challenge represents a novel problem for the future of healthcare. Indeed, healthcare systems are facing shortages of resources of increasing magnitudes; furthermore, the current COVID-19 pandemic underlines the necessity to free up resources as soon as possible, in order to address the continuous and ever-increasing stream of patients that come to hospital, in need of assistance.

### 4.2. Limitations and Strengths

One weakness of this study was the paucity of subjects in Period 2; this may have led to the lack of new findings in the analysis of this time period. One of these missing findings is probably the identification of social issues as factors correlated with the LOS and a difficult discharge. However, as discussed before, age may have included all those aspects as a proxy.

The high number of records analyzed during Period 1 is one of the main strengths of this study, giving relevance to the factors that emerged as statistically significant, and so those factors should be considered.

## 5. Conclusions

Our study suggests that both clinical and social issues (probably masked by the increase in age) have a relevant influence over a longer LOS and the need for additional care after discharge; therefore, those factors should be included in any development tool that tries to identify those patients as early as possible. Furthermore, patients with social vulnerabilities more likely suffer from these if admitted through EDs. While more must be done to reach these patients when they are still not in need of emergency procedures, tools implemented in EDs can help to identify this kind of patient early on, in order to be able to tailor the discharge plan as early as at admission.

## Figures and Tables

**Table 1:** Descriptive statistics.

		Period 1							Period 2
		*n* = 15,165							*n* = 388
Variable		Whole	By Discharge Destination			*p*	LOS ^co^	*p*	Whole
			*Deceased*	*No NAC*	*NAC*				
Age ^co^		62 (22)	78 (16)	61 (22)	71 (21)	<0.001	0.08	<0.001	61 (20)
LOS ^co^		3 (6)	19 (26)	3 (6)	15 (30)	<0.001	-	-	2 (3)
Sex	*Female*	47.7	0.4	97.3	2.3	0.069	3 (6)	0.388	45.9
	*Male*	52.3	0.6	96.7	2.7		3 (7)		54.1
Place of residence	*Same region*	90.4	0.5	97	2.5	0.076	3(6)	0.004	95.4
	*Other region*	9.4	0.2	97.1	2.7		4 (8)		4.6
	*Abroad*	0.2	3.6	92.8	3.6		5 (12)		-
Hospitalization procedure	*Planned*	68.9	0.1	98.3	1.6	<0.001	1 (4)	<0.001	90.2
	*ED*	18.7	1.8	94.1	4.1		7 (10)		1.8
	*Other*	12.4	0.6	94.4	5		6 (10)	<0.001	8
Neoplasia	*Yes*	25.6	1.1	95	3.9	<0.001	7 (13)		17.8
	*No*	74.4	0.3	97.7	2		2 (5)		82.2
Discharge destination	*Deceased*	0.5	-	-	-		-		-
	*No NAC*	97	-	-	-		-		-
	*NAC*	2.5	-	-	-		-		-
DRG—weight > 1	*Yes*	53.1	0.8	95.5	3.7	<0.001	6 (9)	<0.001	-
	*No*	46.9	0.1	98.8	1.1		1 (2)		-
DRG—with complications	*Yes*	18.9	1.9	91.5	6.6	<0.001	9 (12)	<0.001	-
	*No*	81.1	0.2	98.3	1.5		2 (5)		-
Caregiver > 65 y/o or no caregiver	*Yes*	-	-	-	-		-		68.8
	*No*	-	-	-	-		-		31.2
Smoking	*Yes*	-	-	-	-		-		21
	*No*	-	-	-	-		-		79
Living alone	*Yes*	-	-	-	-		-		17.6
	*No*	-	-	-	-		-		82.4
Fall in the preceding year	*Yes*	-	-	-	-		-		10.4
	*No*	-	-	-	-		-		89.6
Walking aid need	*Yes*	-	-	-	-		-		2.9
	*No*	-	-	-	-		-		97.1

LOS: Length of stay. ED: Emergency Department. NAC: Need for additional care after discharge from hospital. DRG: Diagnosis Related Group. Unless otherwise noted, variables are categorical. ^co^ Continuous variable. Figures and statistical tests vary, depending on variable type. Categorical-categorical: relative frequency and chi-square test. Categorical-continuous: median (IQR) and Mann–Whitney U test/Kruskal–Wallis test. Continuous-continuous: unstandardized B and *t*-test, univariable linear regression model.

**Table 2:** Length of stay (LOS) outcomes—linear regression models.

Variable		Period 1—All Variables			Period 1—At Admission			Period 2		
		B	95% CI	*p*	B	95% CI	*p*	B	95% CI	*p*
Age ^co^		0.04	0.03; 0.05	<0.001	0.08	0.07;0.08	<0.001	0.03	0.01; 0.05	0.017
Sex—Female		0.07	−0.19; 0.34	0.585	−0.16	−0.46;0.13	0.273	−0.04	−0.70; 0.62	0.908
Place of residence	*Same region*	Ref.	-	-	Ref.	-	-	Ref.	-	-
	*Other region*	−0.10	−0.56; 0.35	0.661	0.93	0.42;1.43	<0.001	1.11	−0.34; 2.55	0.133
	*Abroad*	−0.77	−4.32; 2.77	0.668	0.41	−3.53;4.36	0.837	-	-	-
Hospitalization procedure	*Planned*	Ref.	-	-	Ref.	-	-	Ref.	-	-
	*ED*	5.21	4.85; 5.57	<0.001	6.92	6.54;7.30	<0.001	8.22	5.94; 10.50	<0.001
	*Other*	4.32	3.90; 4.73	<0.001	6.44	5.99;6.90	<0.001	−0.29	−1.47; 0.89	0.631
Neoplasia presence		2.57	2.24; 2.90	<0.001	-	-	-	3.49	2.65; 4.32	<0.001
Discharge destination	*No NAC*	Ref.	-	-	-	-	-	-	-	-
	*Deceased*	9.78	7.86; 11.70	<0.001	-	-	-	-	-	-
	*NAC*	10.76	9.90; 11.62	<0.001	-	-	-	-	-	-
DRG—weight > 1		4.32	4.01; 4.63	<0.001	-	-	-	-	-	-
DRG—with complications		3.68	3.30; 4.06	<0.001	-	-	-	-	-	-
Caregiver > 65 y/o or no caregiver			-	-	-	-	-	−0.60	−1.32; 0.11	0.099
Smoking			-	-	-	-	-	0.04	−0.74; 0.82	0.925
Living alone			-	-	-	-	-	0.03	−0.80; 0.87	0.935
Fall in the preceding year			-	-	-	-	-	−0.02	−1.05; 1.02	0.969
Walking aid need			-	-	-	-	-	−0.26	−2.20; 1.68	0.792

NAC: Need for additional care. Unless otherwise noted, variables are categorical. ^co^ Continuous variable.

**Table 3:** Discharge destination outcomes—logistic regression models.

Variable		Period 1—All Variables			Period 1—At Admission		
		OR	95% CI	*p*	OR	95%CI	*p*
Age		1.02	1.01; 1.03	<0.001	1.03	1.02;1.03	<0.001
Sex—Female		0.97	0.79; 1.20	0.792	0.92	0.75;1.14	0.445
Place of residence	*Same region*	Ref.	-	-	Ref.	-	-
	*Other region*	0.95	0.67; 1.34	0.783	1.09	0.77;1.53	0.625
	*Abroad*	1.22	0.16; 9.36	0.849	1.47	0.19;11.01	0.710
Hospitalization procedure	*Planned*	Ref.	-	-	Ref.	-	-
	*ED*	1.77	1.37; 2.30	<0.001	2.54	1.99;3.24	<0.001
	*Other*	2.69	2.06; 3.51	<0.001	3.39	2.61;4.40	<0.001
Neoplasia presence		1.24	0.98; 1.56	0.076	-	-	-
DRG—weight > 1		1.65	1.23; 2.22	0.001	-	-	-
DRG—with complications		1.02	1.01; 1.03	<0.001	-	-	-

NAC: Need for additional care. Unless otherwise noted, variables are categorical. ^co^ Continuous variable.

## References

[1] Gonçalves-Bradley D.C., Lannin N.A., Clemson L.M., Cameron I.D., Shepperd S. (2016). Discharge planning from hospital. Cochrane Database Syst. Rev..

[2] Campbell R.C., Dudley H.A. (1964). Hospital stay of patients undergoing minor surgical procedures. Lancet Lond. Engl..

[3] Bithell J.F., Devlin H.B. (1968). Prediction of discharge of hospital inpatients. Health Serv. Res..

[4] Anderson A.D.G., McNaught C.E., MacFie J., Tring I., Barker P., Mitchell C.J. (2003). Randomized clinical trial of multimodal optimization and standard perioperative surgical care. Br. J. Surg..

[5] Morgan M., Beech R. (1990). Variations in lengths of stay and rates of day case surgery: Implications for the efficiency of surgical management. J. Epidemiol. Commun. Health.

[6] De Saint-Hubert M., Schoevaerdts D., Cornette P., D’Hoore W., Boland B., Swine C. (2010). Predicting functional adverse outcomes in hospitalized older patients: A systematic review of screening tools. J. Nutr. Health Aging.

[7] Oldmeadow L.B., McBurney H., Robertson V.J. (2003). Predicting risk of extended inpatient rehabilitation after hip or knee arthroplasty. J. Arthroplast..

[8] Parsonnet V., Dean D., Bernstein A.D. (1989). A method of uniform stratification of risk for evaluating the results of surgery in acquired adult heart disease. Circulation.

[9] Copeland G.P., Jones D., Walters M. (1991). POSSUM: A scoring system for surgical audit. Br. J. Surg..

[10] Zarovska A., Evangelista A., Boccia T., Ciccone G., Coggiola D., Scarmozzino A., Corsi D. (2018). Development and validation of a simplified BRASS index to screen hospital patients needing personalized discharge planning. J. Gen. Intern. Med..

[11] Wiley M.M., Balakrishnan N., Colton T., Everitt B., Piegorsch W., Ruggeri F., Teugels J.L. (2014). Diagnosis related groups (DRGs): Measuring hospital case mix. Wiley StatsRef: Statistics Reference Online.

[12] Robinson G.H., Davis L.E., Leifer R.P. (1966). Prediction of hospital length of stay. Health Serv. Res..

[13] Kobewka D.M., Mulpuru S., Chassé M., Thavorn K., Lavallée L.T., English S.W., Neilipovitz B., Neilipovitz J., Forster A.J., McIsaac D.I. (2020). Predicting the need for supportive services after discharged from hospital: A systematic review. BMC Health Serv. Res..

[14] Department of Health (2004). Achieving Timely ‘Simple’ Discharge from Hospital: A Toolkit for the Multi-Disciplinary Team. http://europepmc.org/guidelines/HIR/59852.

[15] Safavi K.C., Khaniyev T., Copenhaver M., Seelen M., Langle A.C.Z., Zanger J., Daily B., Levi R., Dunn P. (2019). Development and validation of a machine learning model to aid discharge processes for inpatient surgical care. JAMA Netw. Open.

[16] Pecoraro F., Clemente F., Luzi D. (2020). The efficiency in the ordinary hospital bed management in Italy: An in-depth analysis of intensive care unit in the areas affected by COVID-19 before the outbreak. PLoS ONE.

[17] Aceto P., Incalzi R.A., Bettelli G., Carron M., Chiumiento F., Corcione A., Crucitti A., Maggi S., Montorsi M., Pace M.C. (2020). Perioperative management of elderly patients (PriME): Recommendations from an Italian intersociety consensus. Aging Clin. Exp. Res..

[18] Lin H.-S., Watts J.N., Peel N.M., Hubbard R.E. (2016). Frailty and post-operative outcomes in older surgical patients: A systematic review. BMC Geriatr..

[19] George E.L., Arya S. (2019). The importance of incorporating frailty screening into surgical clinical workflow. JAMA Netw. Open.

[20] Havens J.M., Peetz A.B., Do W.S., Cooper Z., Kelly E., Askari R., Reznor G., Salim A. (2015). The excess morbidity and mortality of emergency general surgery. J. Trauma Acute Care Surg..

[21] Achanta A., Nordestgaard A., Kongkaewpaisan N., Han K.R., Mendoza A., Saillant N., Rosenthal M., Fagenholz P., Velmahos G., Kaafarani H. (2019). Most of the variation in length of stay in emergency general surgery is not related to clinical factors of patient care. J. Trauma Acute Care Surg..

[22] McAleese P., Odling-Smee W. (1994). The effect of complications on length of stay. Ann. Surg..

[23] Pirson M., Dehanne F., van den Bulcke J., Leclercq P., Martins D., de Wever A. (2018). Evaluation of cost and length of stay, linked to complications associated with major surgical procedures. Acta Clin. Belg..

[24] Shahrokni A., Tin A., Alexander K., Sarraf S., Afonso A., Filippova O., Harris J., Downey R.J., Vickers A.J., Korc-Grodzicki B. (2019). Development and evaluation of a new frailty index for older surgical patients with cancer. JAMA Netw. Open.

